# Editorial: Enhancing sustainable crop production: biostimulants and biotechnological approaches in challenging climates

**DOI:** 10.3389/fpls.2024.1534774

**Published:** 2024-12-18

**Authors:** Günter Neumann, Fahim Nawaz, Markus Weinmann, Vicent Arbona, Raffaella Balestrini, Chiara Pagliarani, Miguel Gonzalez-Guzman

**Affiliations:** ^1^ Institute of Crop Science, Department Nutritional Crop Physiology, Faculty of Agricultural Sciences, University of Hohenheim, Stuttgart, Germany; ^2^ Research School of Biology, The Australian National University, ACT, Canberra, Australia; ^3^ Laboratori d’Ecofisiologia i Biotecnologia, Departament de Biologia, Bioquímica i Ciències Naturals, Universitat Jaume I, Castelló de la Plana, Spain; ^4^ Institute of Biosciences and Bioresources, National Research Council of Italy, Bari, Italy; ^5^ Institute for Sustainable Plant Protection, National Research Council of Italy, Torino, Italy

**Keywords:** agricultural production systems, efficiency testing, environmental stress protection, green biotechnological strategies, microbial inoculants, non-microbial biostimulants

The implementation of biostimulants (BS; based on plant growth-promoting microorganisms or natural bioactive compounds) as plant strengtheners and other green biotechnological strategies are discussed as promising approaches to cope with increasing challenges for crop production related to climate change, limited availability of resources, and environmental protection. The principle effectiveness of these approaches has been frequently demonstrated, particularly in experiments conducted under controlled conditions, also contributing to a basic understanding of the underlying biological modes of action. However, the poor reproducibility of the expected benefits during field application remains a major challenge in bridging the gap between lab research and practical application, which is a major focus of this Research Topic.

As an initial overview, two review papers addressed various biotechnological approaches currently investigated in this context, including adaptive modification strategies for crops, modification of soil properties, and exploiting interactions with plant-beneficial microbes in different production systems (Melini et al.; Badiyal et al.).

The high variability of responses frequently observed under field conditions suggests a strong impact of environmental factors that can determine the beneficial functions of the respective adaptation strategies. This aspect is addressed by a multilevel approach, starting with three examples of investigations on the modes of action of various non-microbial BS to mitigate environmental stress under controlled conditions. The investigated stress responses comprised protective effects of the plant compound salvianolic acid on osmotic stress in maize and soybean (Kazerooni et al.) as well as mitigation of cold stress and salinity by applications of seaweed extracts and protein hydrolysates in tomato (Borella et al.; Zhang et al.). Metabolomics, transcriptomics, and the analysis of various physiological stress indicators revealed an improved oxidative stress defense as a common mode of action, but also differential effects depending on the type of applied BS products.

At the next level, eight studies presented lab-to-field approaches to test the performance of microbial or non-microbial BS and management practices in different crops under variable environmental conditions. For better understanding of the critical factors interfering with the beneficial effects, it is more insightful to examine not only successful applications but also experiments that failed to produce the expected results. This last aspect was found in three experiments with applications of microbial consortia partially combined with micronutrients, seaweed extracts, and chitosan conducted with maize in Switzerland (Symanczik et al.) or winter rye (Behr et al.) and winter wheat (Gobel et al.) in Germany. In these cases, benefits were observed mainly in pot experiments under controlled conditions (Symanczik et al.; Gobel et al.) and during early growth in field trials (Behr et al.; Symanczik et al.), but did not fully translate into yield effects under field conditions. Conversely, microbial inoculants increased yield and resistance to biotic and abiotic stress factors in field experiments conducted with coffee and black pepper in Vietnam (Thanh Tam et al.), with tomato in Southern Italy (Cirillo et al.), and with maize, in combination with nano zinc fertilization, in Brazil (Jalal et al.). Fruit quality parameters of strawberries in Italy were improved by application of a protein hydrolysate and auxin-rich bacterial filtrates (Cardarelli et al.). Appropriate straw-returning to maize fields in Northern China decreased greenhouse gas emissions and improved the yield potential in maize (Wang et al.).

The third level consists of meta-analyses, which encompass a broad range of studies. This alternative approach offers large-scale insights into potential environmental factors influencing the performance of BS. Recently, various meta-studies have been conducted summarizing research achievements on the different groups of biostimulants (Schütz et al., 2018; Herrmann et al., 2022; Li et al., 2022). However, in a meta-analysis based on already published data, the interpretation of the results may be affected by the so-called “publication bias”, as mainly positive results are usually considered for publication. Conversely, the present Research Topic provides a meta-analysis covering more than 140 pot and field experiments and 107 treatments with microbial and non-microbial BS applied as single products or as product combinations (Nkebiwe et al.). The data set derives from an EU-funded project (BIOFECTOR), investigating the performance of BS in European agriculture. It covers all data generated within the project over five years and is therefore not affected by a publication bias. Accordingly, the reported beneficial BS effects on plant performance with an average growth/yield increase of 9.3% in 945 observations (Nkebiwe et al.) were generally smaller than those reported by meta-studies based on published data (Schütz et al., 2018; Herrmann et al., 2022; Li et al., 2022).

A common outcome of all recently published meta-analyses is an apparent dependence of BS performance on various geo-climatic factors. Two meta-studies covering microbial (Schütz et al., 2018) and non-microbial BS (Li et al., 2022) suggested better performance of BS applications under arid and semiarid or subtropical/tropical climates as compared with more temperate climate conditions. Additionally, three meta-studies on microbial and non-microbial BS (Schütz et al., 2018; Li et al., 2022; Nkebiwe et al.) consistently showed a declining efficiency of BS applications with increasing soil organic matter. Both factors are closely correlated. Temperate climates often pose fewer challenges for crop production because they experience less extreme conditions in temperature, precipitation, soil pH, or salinity. Consequently, there is a reduced need for protective measures such as the application of biostimulants (BS). Moreover, soil organic carbon levels are frequently higher in temperate climates, often associated with higher levels of humic substances, higher fertility, better water-holding capacity, higher microbial activity and diversity, and a higher abundance of beneficial soil biota (Oldfield et al., 2019; Hoffland et al., 2020; Gerke, 2022). This may indicate a higher buffering capacity against the impact of environmental stress factors. In the respective soils, the effects of external BS applications may be at least partially replaced by higher levels of humic substances and native beneficial microbes with similar functions. Accordingly, also in this Research Topic, the absence of beneficial yield effects after BS application was restricted to field experiments conducted under temperate climate conditions in Germany (Behr et al.; Gobel et al.) and Switzerland (Symanczik et al.), while the remaining studies showing positive effects were performed under tropical, subtropical or Mediterranean climates (Thanh Tam et al.; Cirillo et al; Jalal et al.; Wang et al.).

For microbial inoculants, Symanczik et al. highlighted the importance of root colonization and rhizosphere competence for the establishment of beneficial effects, which was sufficient in controlled greenhouse studies during the early growth of maize but rapidly declined under field conditions. This is in line with the meta-analysis carried out by Nkebiwe et al., showing better field performance after BS application in crops maintained in a protected nursery before transplanting to the field compared with BS inoculation performed directly under field conditions.

Improved performance of microbial inoculants in combination with manure-based organic fertilizers in comparison with mineral fertilization was reported by Behr et al., similar to various previously published studies (Thonar et al., 2017; Mpanga et al., 2018; Bradáčová et al., 2019) and the meta-analysis by Nkebiwe et al. in this Research Topic. The application of organic fertilizers with easily available carbon sources might improve the carbon supply for fast-growing copiotrophic inoculants as well as indigenous plant growth-promoting microorganisms and support the establishment of a beneficial microbial community (Behr et al.). Furthermore, the high availability of N and P in manure-based fertilizers may serve as a starter fertilization for the host plant, facilitating root growth and the establishment of microbial inoculants in the rhizosphere (Bittman et al., 2006; Chekanai et al., 2018).

All the meta-studies cited here highlighted genotypic differences at the plant species level as key factors influencing BS) interactions with host plants. These differences may stem from variations in compatibility, as well as differences in growing conditions (Nkebiwe et al.), the severity and timing of imposed stress conditions, and/or variability in stress tolerance of different plant varieties (Mahmood et al., 2022). Seven studies of this Research Topic used BS combinations (Behr et al.; Cirillo et al.; Gobel et al.; Jalal et al.; Mendes et al.; Symanczik et al.; Zhang et al.), frequently employed as a strategy to provide higher flexibility under variable environmental conditions (Nuti and Giovannetti, 2015; Sekar et al., 2016; Furlan et al., 2019). This was confirmed by the meta-study of Herrmann et al. (2022). However, the benefits of BS combinations were preferentially observed under stress conditions (Bradáčová et al., 2019; Nkebiwe et al.), and increased the propability of beneficial effets but not necessarily the absolute effect size (Bradáčová et al., 2019; Mamun et al., 2024).

Three studies pointed out the importance of interactions of microbial inoculants with native soil-microbial communities for the expression of beneficial BS effects in different crop species (Behr et al.; Cirillo et al.; Mendes et al.) as an aspect that deserves particular attention in future BS research, together with the impact on different genotypes inside a species. Finally, methodological difficulties related to the efficiency testing of BS-assisted strategies and green-biotechnological approaches were addressed by Mendes et al., Neuhoff et al., and Sun et al.

Collectively, the articles included in this Research Topic offer diverse examples for critical evaluation and characterization of conditions promoting the development of integrated plant production systems supported by environmentally friendly approaches based on BS applications and other green biotechnological strategies.

## References

[B1] Bittman S. Kowalenko C. G. Hunt D. E. Forge T. A. Wu X. (2006). Starter phosphorus and broadcast nutrients on corn with contrasting colonization by mycorrhizae. Agron. J. 98, 394–401. doi: 10.2134/agronj2005.0093

[B2] Bradáčová K. Florea A. S. Bar-Tal A. Minz D. Yermiyahu U. Shawahna R.. (2019). Microbial consortia versus single-strain inoculants: an advantage in PGPM-assisted tomato production? Agronomy 9, 105. doi: 10.3390/agronomy9020105

[B3] Chekanai V. Chikowo R. Vanlauwe B. (2018). Response of common bean (Phaseolus vulgaris L.) to nitrogen, phosphorus and rhizobia inoculation across variable soils in Zimbabwe. Agric. Ecosyst. Environ. 266, 167–173. doi: 10.1016/j.agee.2018.08.010 30393414 PMC6142818

[B4] Furlan A. Bianucci E. Sequeira M. Álvarez L. Peralta J.M. Valente C. (2019). “Combined application of microbial and non-microbial biostimulants to improve growth of peanut plants exposed to abiotic stresses,” in Microbial probiotics for agricultural systems. Sustainability in plant and crop protection. Eds. Zúñiga-Dávila D. González-Andrés F. Ormeño-Orrillo E. (Springer, Cham). doi: 10.1007/978-3-030-17597-9_17

[B5] Gerke J. (2022). The central role of soil organic matter in soil fertility and carbon storage. Soil Syst. 6, 33. doi: 10.3390/soilsystems6020033

[B6] Herrmann M. N. Wang Y. Hartung J. Hartmann T. Zhang W. Nkebiwe P. M.. (2022). A global network meta-analysis of the promotion of crop growth, yield, and quality by bioeffectors. Front. Plant Sci. 13. doi: 10.3389/fpls.2022.816438 PMC8921507 35300013

[B7] Hoffland E. Kuyper T.W. Comans R.N.J Craemer R.E.. (2020). Eco-functionality of organic matter in soils. Plant Soil 455, 1–22. doi: 10.1007/s11104-020-04651-9

[B8] Li J. Van Gerrewey T. Geelen D. (2022). A meta-analysis of biostimulant yield effectiveness in field trials. Front. Plant Sci. 13. doi: 10.3389/fpls.2022.836702 PMC9047501 35498677

[B9] Mahmood A. Goertz S. Moradtalab N. Walker F. Höglinger B. Ludewig U.. (2022). Drought-protective effects of nutrient seed treatments during early growth of oilseed rape. J. Plant Nutr. 45, 2945–2963. doi: 10.1080/01904167.2022.2067049

[B10] Mamun A. A. Neumann G. Moradtalab N. Ahmed A. Dupuis B. Darbon G.. (2024). Microbial consortia versus single-strain inoculants as drought stress protectants in potato affected by the form of N supply. Horticulturae 10, 102. doi: 10.3390/horticulturae10010102

[B11] Mpanga I. K. Dapaah H. K. Geistlinger J. Ludewig. U. Neumann G. (2018). Soil type-dependent interactions of P-solubilizing microorganisms with organic and inorganic fertilizers mediate plant growth promotion in tomato. Agronomy 8, 213. doi: 10.3390/agronomy8100213

[B12] Nuti M. Giovannetti G. (2015). Borderline products between bio-fertilizers/bio-effectors and plant protectants: the role of microbial consortia. J. Agric. Sci. Technol. A 5, 305–315. doi: 10.17265/2161-6256/2015.05.001

[B13] Oldfield E. E. Bradford M. A. Wood S. A. (2019). Global meta-analysis of the relationship between soil organic matter and crop yields. Soil 5, 15–32. doi: 10.5194/soil-5-15-2019

[B14] Schütz L. Gattinger A. Meier M. Müller A. Boller T. Mäder P.. (2018). Improving crop yield and nutrient use efficiency via biofertilization - a global meta-analysis. Front. Plant Sci. 8. doi: 10.3389/fpls.2017.02204 PMC5770357 29375594

[B15] Sekar J. Raj R. Prabavathy V. R. (2016). “Microbial consortial products for sustainable agriculture: commercialization and regulatory issues in India,” in Agriculturally important microorganisms. Eds. Singh H. B. Sarma B. K. Keswani C. (Springer Science+Business Media, Singapore), 107–131.

[B16] Thonar C. Lekfeldt J. D.S. Cozzolino V. Kundel D. Kulhánek M. Mosimann C. (2017). Potential of three microbial bio-effectors to promote maize growth and nutrient acquisition from alternative phosphorous fertilizers in contrasting soils. Chem. Biol. Technol. Agric. 4, 7. doi: 10.1186/s40538-017-0088-6

